# Tapioca Resistant Maltodextrin as a Carbohydrate Source of Oral Nutrition Supplement (ONS) on Metabolic Indicators: A Clinical Trial

**DOI:** 10.3390/nu14050916

**Published:** 2022-02-22

**Authors:** Junaida Astina, Weeraya Saphyakhajorn, Chaleeda Borompichaichartkul, Suwimol Sapwarobol

**Affiliations:** 1 Graduate Program in Food and Nutrition, Department of Nutrition and Dietetics, Faculty of Allied Health Sciences, Chulalongkorn University, Bangkok 10330, Thailand; junaida.astina@gmail.com; 2 The Medical Food Research Group, Department of Nutrition and Dietetics, Faculty of Allied Health Sciences, Chulalongkorn University, Bangkok 10330, Thailand; weeraya.b@outlook.com; 3 Department of Food and Technology, Faculty of Sciences, Chulalongkorn University, Pathum Wan, Bangkok 10330, Thailand; chaleeda.b@chula.ac.th

**Keywords:** glucose, gastrointestinal tolerability, insulin, oral nutritional supplement, tapioca resistant maltodextrin

## Abstract

Tapioca resistant maltodextrin (TRM) is a novel non-viscous soluble resistant starch that can be utilized in oral nutrition supplements (ONS). This study aims to evaluate acute and long-term metabolic responses and the safe use of ONS containing TRM. This study comprised of two phases: In Phase I, a randomized-cross over control study involving 17 healthy adults was conducted to evaluate three ONS formulations: original (tapioca maltodextrin), TRM15 (15% TRM replacement), and TRM30 (30% TRM replacement). Plasma glucose, serum insulin, and subjective appetite were evaluated postprandially over 180 min. In Phase II, 22 participants consumed one serving/day of ONS for 12 weeks. Blood glucose, insulin, lipid profile, and body composition were evaluated. Gastrointestinal tolerability was evaluated in both the acute and long-term period. During phase I, TRM30 decreased in area under the curve of serum insulin by 33.12%, compared to the original formula (2320.71 ± 570.76 uIU × min/mL vs. 3470.12 ± 531.87 uIU × min/mL, *p* = 0.043). In Phase II, 12-week TRM30 supplementation decreased HbA1C in participants (from 5.5 ± 0.07% to 5.2 ± 0.07%, *p* < 0.001), without any significant effect on fasting glucose, insulin, lipid profile, and body composition. The ONS was well-tolerated in both studies. TRM is therefore, a beneficial functional fiber for various food industries.

## 1. Introduction

Tapioca resistant maltodextrin (TRM) is a novel resistant starch, type IV, produced from tapioca starch. It is produced by debranching the tapioca starch structure using enzymatic hydrolysis, leaving the α,1-2, α,1-3, and other linkages indigestible by human digestive enzymes. The glycemic index evaluation of TRM is 59 for healthy subjects, which increases the blood glucose slower than digestible maltodextrin [1]. Tapioca-resistant maltodextrin contains 180 kcal/100 g and carbohydrate 94 g/100 g [2]. Due to its non-viscous texture, neutral flavor, and lack of color, TRM is considered a functional ingredient in various healthy food products [3]. Studies reported that incorporating resistant starch into food and beverages improved glycemic response [4], insulin sensitivity [5], triglyceride reduction [6], weight control [7], and appetite regulation [8]. In a previous study, 50 g of TRM significantly reduced the peak of postprandial blood glucose among healthy individuals when compared to 50 g of digestible tapioca maltodextrin (TM) and 50 g of glucose by 18% and 23%, respectively [9]. In addition, consumption of 34 g wheat-resistant maltodextrin daily for 12 weeks benefited glycemic control by improving plasma glucose, insulin, and HOMA-IR in overweight men [5].

Currently, several starches have been utilized to produce resistant maltodextrin, such as corn, wheat, potato, and tapioca starch. Different sources of resistant maltodextrin may have different starch digestibility due to their different intrinsic and extrinsic factors [9]. A previous study showed 5 g of potato resistant maltodextrin was able to lower postprandial plasma glucose in 10 healthy males [10]. In contrast, 16 g of corn starch maltodextrin did not significantly reduce postprandial blood glucose in 30 healthy adults [11].

Foods for Special Medical Purposes (FSMPs) are intended for exclusive or partial use for malnourished patients or patients with impaired capacity to ingest, digest, or absorb nutrition from a normal diet. These foods include oral nutrition supplement (ONS), which can be disease specific. Complete standard nutritional ONS contains macro and micronutrients as recommended by recommended daily intake (RDI); therefore, they can be used for the long term [12]. FSMPs are formulated to provide sufficient energy and macro (carbohydrate, protein, and fat) and micronutrients (vitamin and minerals) at different amounts according to the patients’ varying medical conditions. Several types of ONS are commercially available, and corn-starch maltodextrin is usually added as a carbohydrate source [13,14]. Based on available literature, the potential use of TRM as a carbohydrate source in novel ONS without negative effects on glycemic and insulin response has been hypothesized. Thus, this study aimed to investigate metabolic indicators, including glycemic and insulin response, using two novel ONS formulas containing 15% and 30% TRM. In addition, acceptance (GI tolerance and palatability) and satiety of the novel ONS were also determined. Metabolic indicators of long-term (12 weeks) use of the novel ONS were also evaluated in healthy and prediabetic participants.

## 2. Materials and Methods

### 2.1. Oral Nutritional Supplements Composition

TM (Caleen-D19), TRM (Cal DM), whey protein isolate, and soy protein isolate (Banpong Novitat, Co., Ltd., Bangkok, Thailand) were used as carbohydrate and protein sources. Blended omega-3 oil powder (Ming Chyi Biotechnology Ltd., Yunlin County, Taiwan) and rice bran creamer (Thai Edible Oil Co., Ltd., Bangkok, Thailand) were used as a source of fat. Vitamin and mineral premixes (DSM Nutritional Products Malaysia Sdn Bhd., Selangor, Malaysia) were added as a source of vitamins and minerals. The macronutrient composition of the three formulas is shown in Table 1. All formulas contained carbohydrate, protein, and fat by 52:16:32. Except for TM and TRM compositions, the three formulas contained identical amounts of other ingredients. Vitamins and minerals contained in ONS were added as recommended by the Thai DRI. The percentage of carbohydrate source for the three formulas was: original formula (TM 78.26% and sucrose 21.74%), TRM15 formula (TM 66.53%, TRM 11.74%, and sucrose 21.74%), and TRM30 formula (TM 54.78%, TRM 23.48%, and sucrose 21.74%). In this study, the original formula was used as a control. One serving of original ONS (56 g powder) provides 252 kcal, 32.7 g carbohydrate, 9.8 g protein, and 9.0 g fat. One serving of TRM15 (56 g) contains 246 kcal, 32.7 g carbohydrate, 9.8 g protein, and 9.0 g fat, while a serving of TRM30 contains 241 kcal, 32.7 g carbohydrate, 9.8 g protein, and 9.0 g fat.

### 2.2. Study Design

#### 2.2.1. Phase I: Acute Effect of Three Developed ONS on Blood Glucose, Insulin Responses, Subjective Appetite, Sensory Acceptability, and Gastrointestinal (GI) Tolerability

##### Study Protocol

This randomized, single blinded, cross-over controlled trial was conducted at the Nutrition and Dietetics Department, Faculty of Allied Health Sciences, Chulalongkorn University. Eligible participants were randomized into three groups (original, TRM15, and TRM30) by an online random sampling generator (Research Randomizer, www.randomizer.org, accessed on 1 March 2021). The inclusion criteria were healthy adults aged 18 to 55, with fasting plasma glucose less than 100 mg/dL and body mass index (BMI) less than 23.0 kg/m^2^. Those who used any medications/insulin injection/herbal supplements to lower plasma glucose or had any allergies to the products used in the study were excluded. Twenty-one participants were screened, and seventeen participants met the inclusion criteria. On the clinic day, one serving (56 g powder) of each ONS formula was dissolved in 200 mL distilled water and mixed until completely dissolved.

Participants fasted overnight (10 h) prior to the clinic day. Upon arrival, the baseline body weight, blood samples, and satiety measurements were taken. A registered nurse inserted the IV catheter into the antecubital vein, drew the baseline blood specimen, and 10 mL of normal saline solution (0.09%) was used to flush the catheter to prevent blood clotting. Then participants were requested to drink the assigned ONS within two minutes. Subsequently, blood samples, and subjective appetite was measured at 30, 60, 120, and 180 min following ONS consumption. Participants were seated and relaxed during the test. A liter of drinking water was allowed at 180 min.

##### Blood Sample Analysis

Blood samples were collected in two types of tubes: sodium fluoride containing tubes for plasma glucose analysis and gel clot activator tubes for serum insulin analysis. Blood samples were centrifuged at 3000 rpm for 10 min and analyzed. Plasma glucose was analyzed by hexokinase method using a clinical chemistry analyzer (Beckman Coulter AU480, Brea, CA, USA). Serum insulin was determined by chemiluminescent microparticle immunoassay (CMIA).

##### Subjective Appetite Measurement

Subjective appetite was measured by using a visual analogue scale (VAS). There were four questions related to appetite, including, “How hungry are you right now?”, “How satiated are you right now?”, “How strong is your desire to eat right now?”, and “How much you can eat right now?” [15]. Participants rated their appetite on a 100 mm line scale with two opposite words anchored at each end (very weak–very strong) [15].

##### Sensory Acceptability

The acceptability of developed ONS was evaluated by using a 9-point hedonic scale [16]. Participants were asked to rate their acceptance of ONS attributes where 9 represented “like extremely” to 1 which represented “dislike extremely”. There were six attributes, including appearance, taste, smell, viscosity, aftertaste, and overall acceptability.

##### Gastrointestinal (GI) Tolerability

The GI tolerability of the developed ONS was evaluated using a GI symptoms questionnaire. There were five symptoms evaluated: abdominal pain, nausea, vomiting, bloating, and flatulence. Participants should rate the intensity of the symptoms (0 = none, 1 = mild, 2 = moderate, 3 = severe) 24 h following ONS consumption [17]. Participants could write any details of the symptoms (e.g., time, duration, or detailed feelings) in the comment box. In addition, the Bristol Stool Scale was used to evaluate the stool form of participants [18]. Evaluation of gastrointestinal symptoms was by questionnaire and the Bristol Stool Scale stool form, 24 h following the ONS consumption.

##### Statistical Analysis

Incremental plasma glucose was calculated as difference of blood glucose at each time point from the fasting baseline blood glucose [19]. The incremental area under the curve was obtained by calculating the area over the baseline under the curve following the trapezoidal rule while ignoring the area below the baseline [20].

$$AUC=\overset{x=1}{\underset{n}{\sum }}Ax$$

Ax is the area under the curve for the *x*th time interval [20].

Data were expressed as mean with standard error of mean. All statistical analysis was performed using Statistical Package for Social Sciences version 22.0 (IBM Corp, Armonk, NY, USA). The Shapiro–Wilk test was performed to analyze the normality of the data. Repeated-measures analysis of variance (RM ANOVA) was carried out to analyze the normally distributed data, while Friedman’s two-way analyze of variance by rank was carried out for non-normally distributed data analysis. Significant value was set at 0.05. Sample size was determined to detect 30% mean difference of plasma glucose with a significance level of 0.05 and 80% power [21]. We hypothesized that TRM-containing ONS would decrease the plasma glucose; therefore, we chose plasma glucose as the primary indicator in sample size calculation. A total of 13 participants were required to meet the minimum sample size.

#### 2.2.2. Phase II: Long-Term (12 Weeks) Effect of Selected ONS on Blood Lipid Profile, Gastrointestinal (GI) Tolerability, and Dietary Intake

##### Study Protocol

This single arm prospective study was conducted at the Nutrition and Dietetics Department, Faculty of Allied Health Sciences, Chulalongkorn University. Eligible participants consume one serving (56 g) of TRM30 daily for 12 weeks on their convenient time. The inclusion criteria of the study were adults aged 18 to 55 who had fasting plasma glucose less than 126 mg/dL. Those who were pregnant or breastfeeding, used any medications/insulin injection/herbal supplements to lower plasma glucose, or had any allergies to the study product were excluded from this study. Eighty-four participants were screened, and twenty-eight met inclusion criteria. Participants were invited to come to the Nutrition and Dietetics Department so blood specimens could be taken; in addition, body composition, dietary intake, physical activity, and GI symptoms at baseline were recorded at week 0, week 3, 6, 9, and 12. On every clinic visit, participants were given 21 sachets of ONS for every 21 days. Compliance was evaluated by counting the empty sachets. Telephone calls twice per week were also made to improve compliance.

##### Blood Sample Analysis

Blood samples were collected in three types of tubes: sodium fluoride containing tubes for plasma glucose analysis, gel clot activator tubes for serum insulin, lipid profile, creatinine, BUN, AST, and ALT analysis, and a tube containing ethylenediaminetetraacetic acid (EDTA) for HbA1C analysis. Blood samples were centrifuged at 3000 rpm for 10 min and analyzed. Fasting plasma glucose (FPG) was analyzed by hexokinase method using clinical chemistry analyzer (Beckman Coulter AU480, Brea, CA, USA). Serum insulin was determined by chemiluminescent microparticle immunoassay (CMIA). Cholesterol and triglyceride were evaluated using the enzymatic method, high-density lipoprotein (HDL)-cholesterol was evaluated by using a homogenous technique, while low-density lipoprotein (LDL) was calculated using Friedewald’s formula [22]. Liver function enzymes, including aspartate transaminase (AST) and alanine transaminase (ALT), were analyzed using the NADH method. Hemoglobin A1C (HbA1C) was evaluated using the enzymatic method. Blood Urea Nitrogen (BUN) and creatinine were evaluated enzymatically, while estimated glomerulus filtration rate was calculated following a CKD-EPI equation [23].

##### Gastrointestinal (GI) Tolerability

The GI tolerability of the developed ONS was evaluated using a GI symptoms questionnaire. There were five symptoms evaluated, including abdominal pain, nausea, vomiting, bloating, and flatulence. Participants should rate the intensity of the symptoms (0 = none, 1 = mild, 2 = moderate, 3 = severe) 24 h following ONS consumption [17]. Participants could write any details of the symptoms (e.g., time, duration, or detailed feelings) in the comment box. In addition, the Bristol Stool Scale was used to evaluate the stool form of the participants [18]. Evaluation of gastrointestinal symptoms was by questionnaire and the Bristol Stool Scale stool form, 24 h following the ONS consumption.

##### Dietary Intake

Dietary intake was evaluated by using dietary record 3 × 24 h (two weekdays and one day over the weekend). Type and amounts of dietary intake and the cooking method were recorded and analyzed by INMUCAL, a Thai nutrients composition program [24].

##### Physical Activity

Physical activity was evaluated by using the Global Physical Activity Questionnaire (GPAQ) [25]. Physical activity is divided into three domains, including activity at work, travel to and from places, and recreational activities. Participants were asked about the type (vigorous-moderate intensity of work and recreation) and duration of the activities. In addition, duration of sedentary activity was also recorded. Physical activity data was expressed as MET.min/week.

##### Statistical Analysis

The Shapiro–Wilk test was used to test the normality of data. A paired samples test was used to analyze the normally distributed data, while non-normally distributed data of blood chemistry and body composition were analyzed by using the Wilcoxon-signed rank test to compare data at week 0 and week 12. Repeated measures analysis of variance (RM-ANOVA) was performed to analyze normally distributed data, while Friedman’s two-way ANOVA by ranks was used to analyze the non-normally distributed data of dietary intake, GI symptoms, and physical activity to compare data at week 0, 3, 6, 9, and 12. All statistical analysis was performed using Statistical Package for Social Sciences version 22.0 (IBM Corp, Armonk, NY, USA). *p*-value less than 0.05 was considered as statistically significant. A minimum of 18 participants was required to evaluate the significance on fasting plasma glucose with effect size of 0.7083, level of significance at 0.05, and power was set at 80% [26]. Sample size was estimated using G* power version 3.1 [27]. Dropout rate was estimated at 30%; thus, 24 participants were recruited in this study.

### 2.3. Ethics

Both phases were approved by the Research Ethics Review Committee for Research Involving Human Subjects (No. 196/60), Health Science Group, Chulalongkorn University, Bangkok, Thailand. All participants were informed about the details of the study, including the risks, benefits, and procedure of the study. Participants signed the informed consent form before being included into the study. Personal data of participants were kept confidential. This study was registered at Thai Clinical Trials with registry number TCTR20210330005 (http://www.thaiclinicaltrials.org/, accessed on 30 March 2021).

## 3. Results

### 3.1. Phase I: Acute Effects of Three Developed ONS on Blood Glucose, Insulin Responses, Subjective Appetite, Sensory Acceptability, and Gastrointestinal (GI) Tolerability

**Figure 1:**
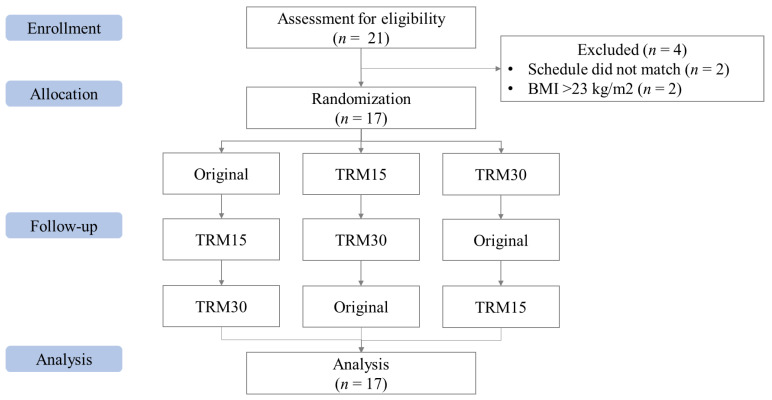
CONSORT flow of the Phase I study.

In total, 21 participants were screened for eligibility. However, two participants were excluded due to their BMI being greater than 23.0 kg/m^2^ and two more due to conflicting schedules, leaving 17 participants (Figure 1). Most of the participants were female (58.8%), mean age 26 ± 0.62 years old. The mean body weight and BMI were 56.05 ± 2.02 kg and 21.29 ± 0.50 kg/m^2^ (Table 2).

#### 3.1.1. Postprandial Plasma Glucose and Insulin Response

**Figure 2:**
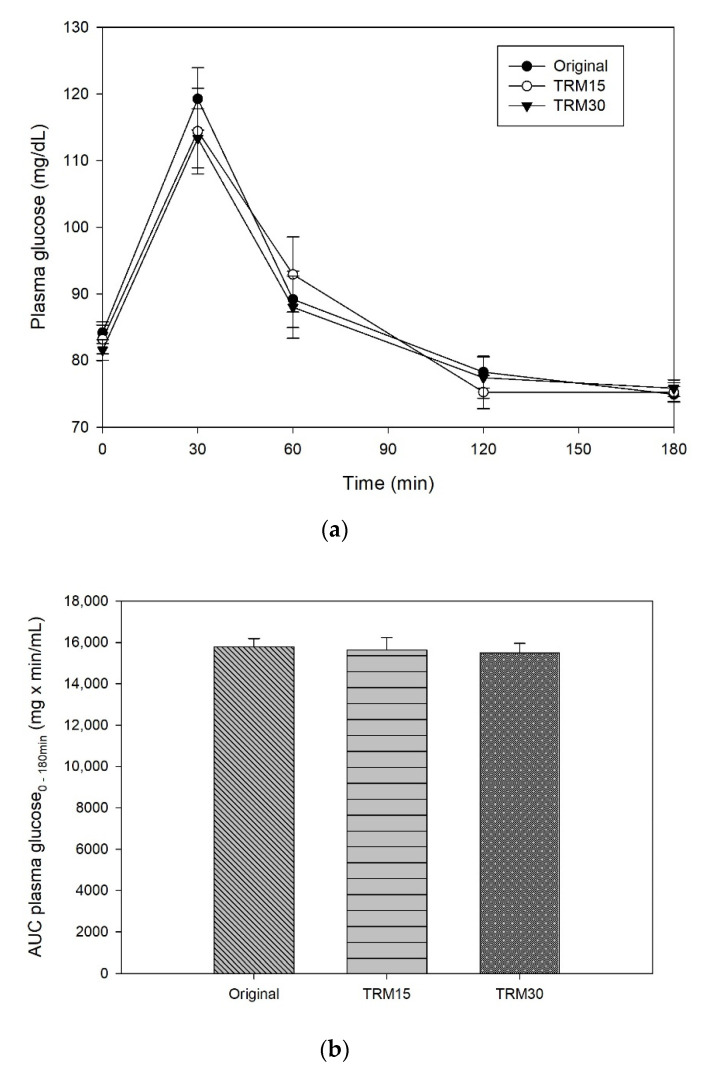

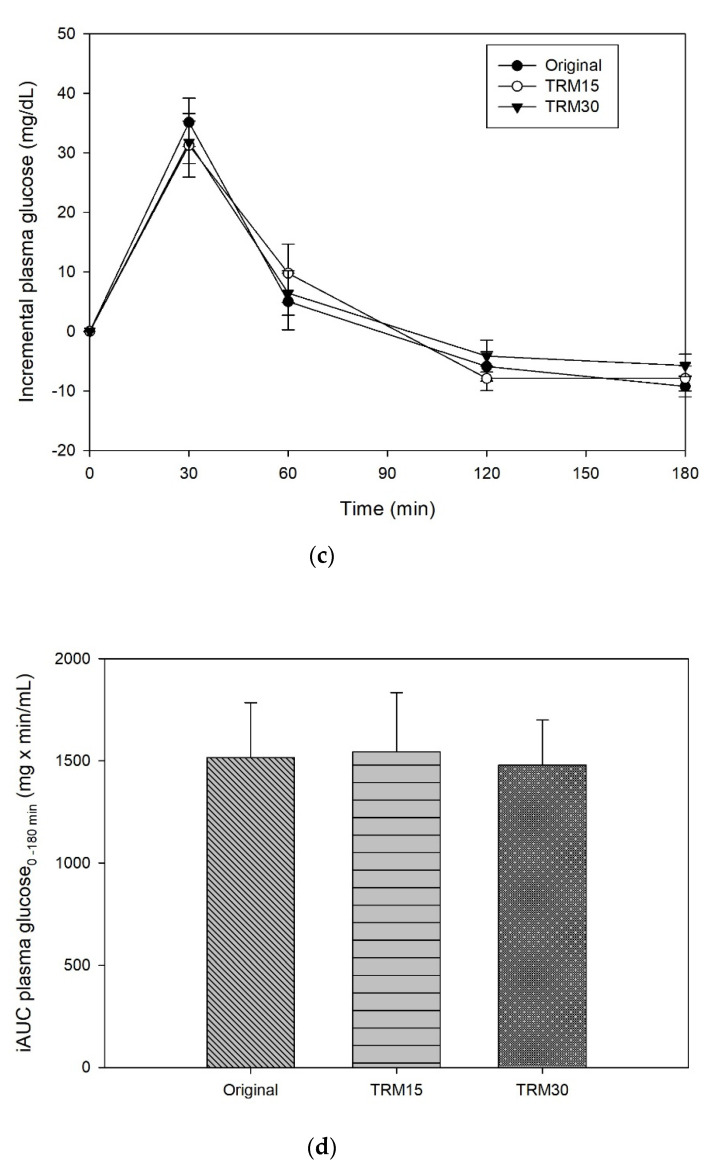
(**a**) Postprandial plasma glucose, (**b**) AUC glucose 0–180 min, (**c**) incremental plasma glucose, and (**d**) iAUC glucose 0–180 min following tapioca RMD-modified ONS.

The peaks of plasma glucose were reached at 30 min following all formula ONS consumptions (Figure 2). The postprandial plasma glucose concentration at 30 min of TRM30 was lowest (113.33 ± 4.44 mg/dL), compared with those after TRM15 (114.42 ± 6.43 mg/dL) and the original formula (119.25 ± 4.63 mg/dL); (to convert glucose in mg/dL to mmol/l, multiply by 0.0555.). Replacement by TRM 30% and 15% decreased postprandial plasma glucose by 4.96% and 4.05%, respectively (*p*-value treatment × time = 0.473). The peak of incremental plasma glucose (0–180 min) following TRM30, TRM15, and original formulas were 31.75 ± 3.54 mg/dL, 31.25 ± 5.37 mg/dL, and 35.08 ± 4.06 mg/dL, respectively. Replacement of TRM by 30% and 15% reduced the peak of incremental plasma glucose by 9.50%, and 10.93% respectively. In addition, incremental AUC (iAUC) of plasma glucose of the TRM30, TRM15, and original formulas were 1480 ± 219.94, 1543.75 ± 290.56 and 1515 ± 269.59 mg × min/dL, respectively (*p* = 0.970). However, those reductions were not significant.

**Figure 3:**
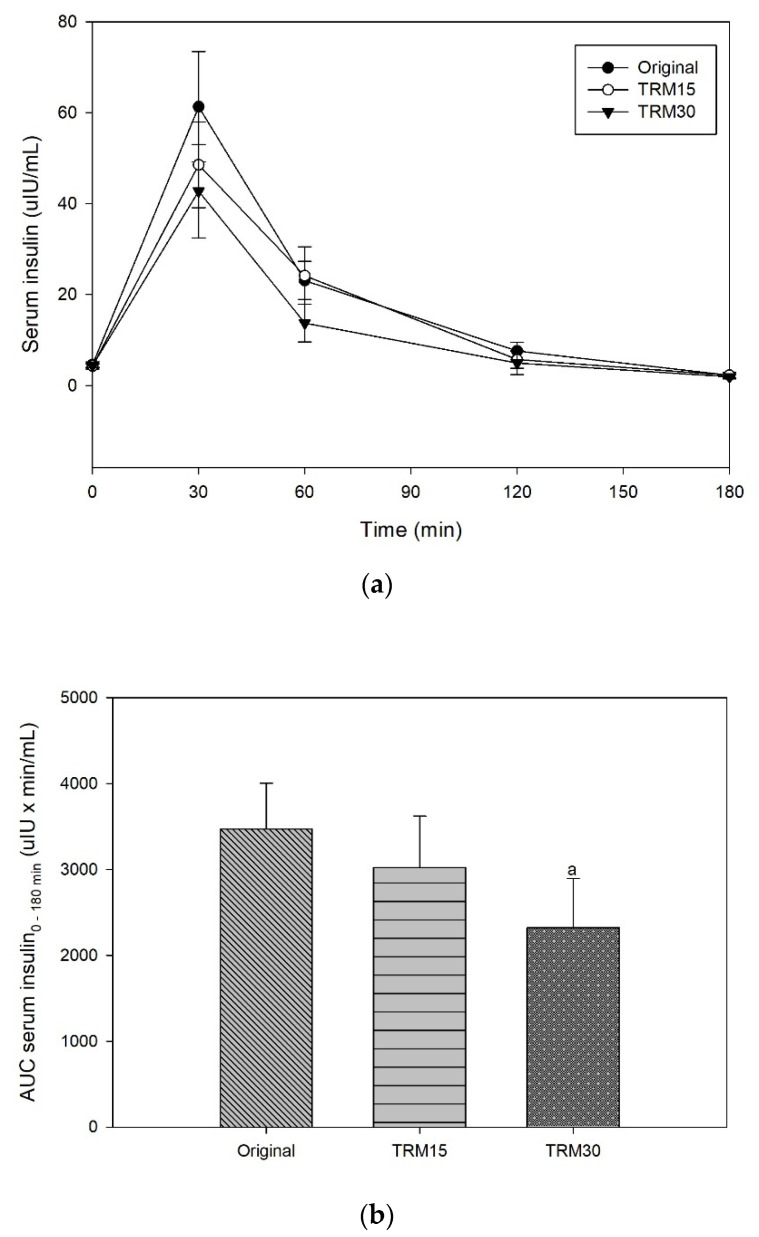

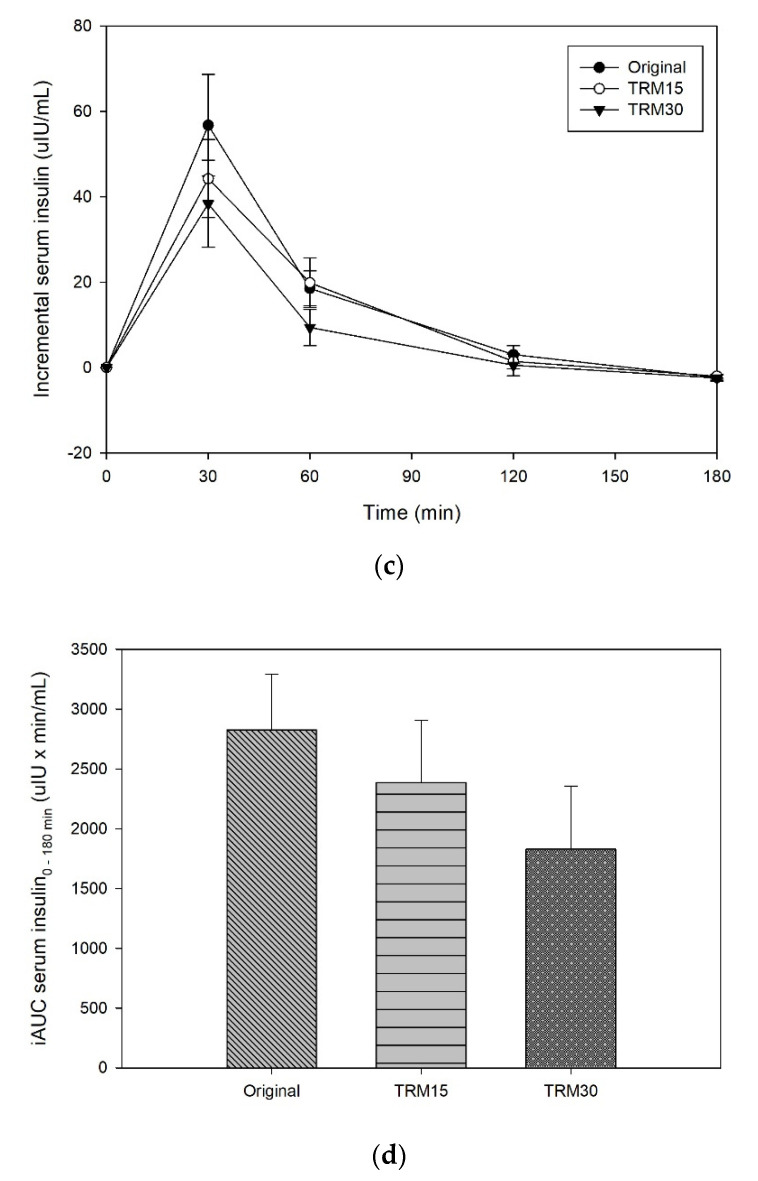
(**a**) Postprandial serum insulin, (**b**) AUC insulin 0–180 min, (**c**) incremental serum insulin, and (**d**) iAUC insulin 0–180 min following tapioca RMD-modified ONS in healthy adults.

At the baseline, there was no significant difference in postprandial serum insulin among participants for every ONS formula. The peaks of serum insulin following each ONS consumption were reached at 30 min in all formulas (Figure 3). The insulin peak of TRM30 was lowest (42.74 ± 10.24 µIU/mL), followed by TRM15 (48.53 ± 9.41 µIU/mL), and original ONS (61.30 ± 12.14 µIU/mL). The incremental serum insulin (0–180 min) following original ONS was 56.72 ± 11.92 µIU/mL, while replacement of tapioca maltode×trin using TRM 30% and 15% decreased the peak of insulin to 38.35 ± 10.23 µIU/mL (32.39%) and 44.22 ± 9.14 µIU/mL (22.05%), respectively. The area under the curve of serum insulin over 180 min following TRM30, TRM15, and the original formula were 2320.71 ± 570.76 µIU × min/mL, 3020.10 ± 600.17 µIU × min/mL, and 3470.12 ± 531.86, respectively. Meanwhile, TRM30 decreased AUC serum insulin over 180 by 33.12%, and TRM15 by 12.97%, respectively (*p* = 0.039). Incremental AUC insulin following TRM30, TRM15, and the original formula were 1827.31 ± 528.49 µIU × min/mL, 2386.05 ± 520.68 µIU × min/mL, and 2825.64 ± 468.24 µIU × min/mL, respectively.

#### 3.1.2. Subjective Appetite

**Figure 4:**
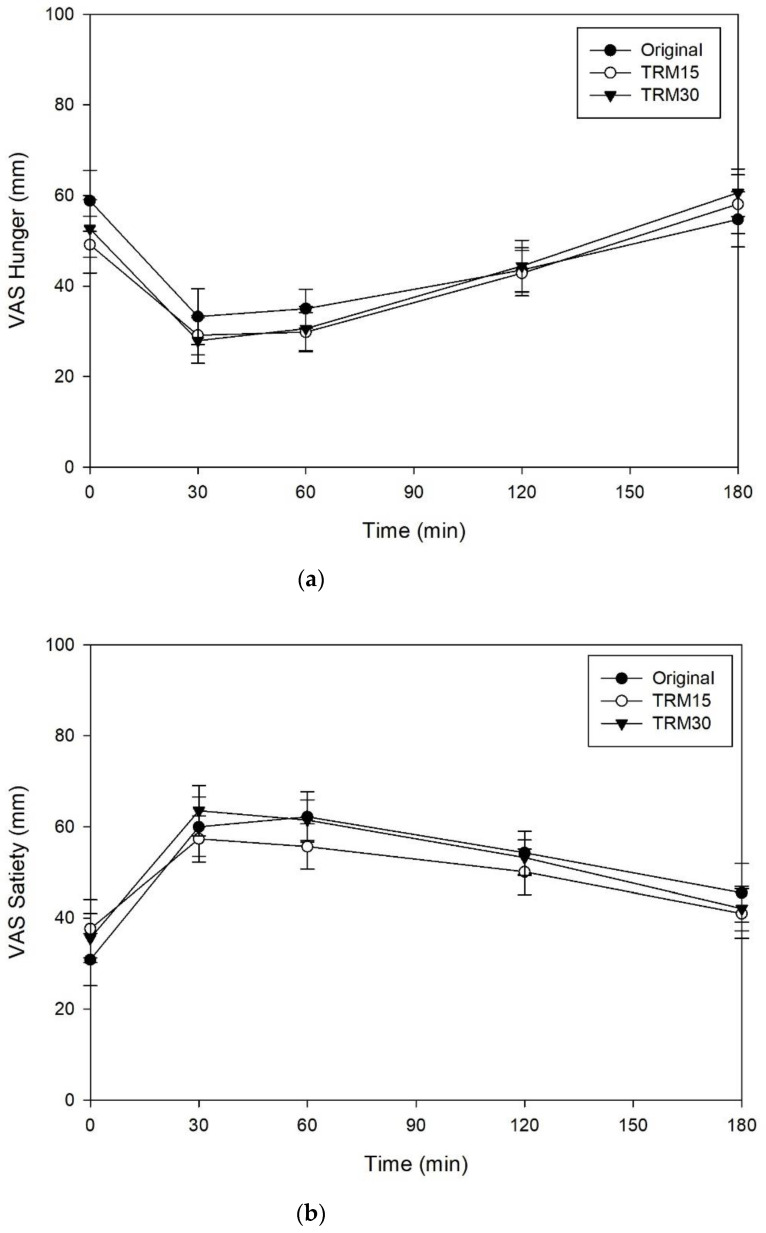

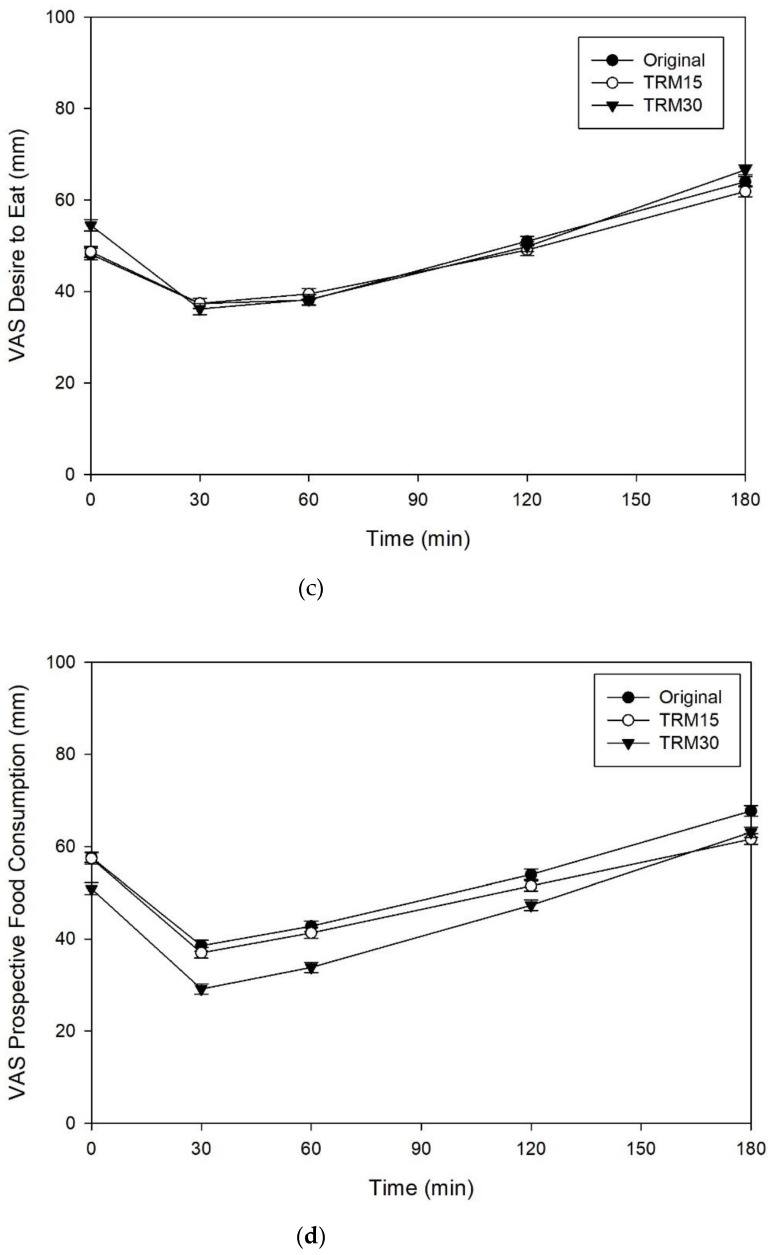
(**a**) Subjective appetite ratings, including hunger, (**b**) satiety, (**c**) desire to eat, and (**d**) prospective food consumption following tapioca RMD-modified ONS in healthy adults.

The effects of ONS consumption on hunger, satiety, desire to eat, and prospective food consumption are shown in the Figure 4. There were no significant differences between ONS formulas for hunger, satiety, desire to eat, and prospective food consumption (*p* > 0.05).

#### 3.1.3. Sensory Acceptability

**Figure 5:**
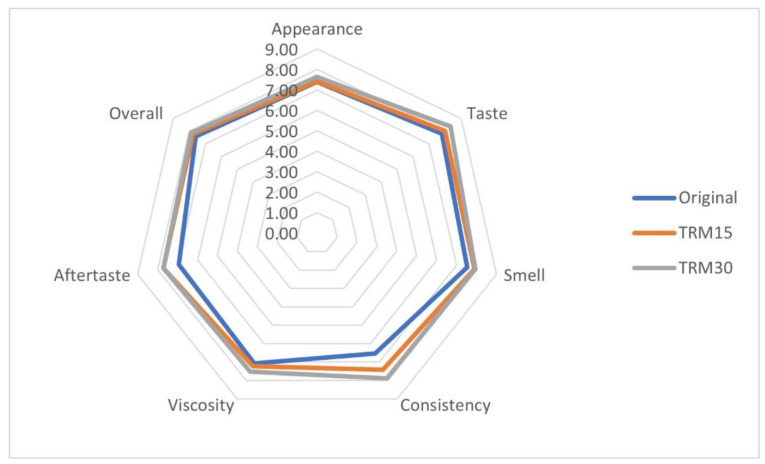
Sensory acceptability of the developed ONS.

The overall acceptability of the TRM30, TRM15 and original formulas were 7.87 ± 0.15, 7.69 ± 0.15 and 7.60 ± 0.23, respectively (Figure 5). Participants rated higher scores of all sensory parameters for TRM30 formula, particularly for viscosity, when compared to the original formula (8.00 ± 0.20 vs. 6.54 ± 0.41, *p* = 0.06).

#### 3.1.4. Gastrointestinal Tolerability

According to gastrointestinal tolerability evaluation, one of 17 participants experienced abdominal pain following TRM30 consumption. No nausea was reported following all ONS formulas. However, mild vomiting was reported for TRM30 (*n* = 1; 5.88%) and TRM15 (*n* = 1; 5.88%). Bloating was reported for the original formula (*n* = 1; 5.88%) and TRM30 formula (*n* = 1; 5.88%). Flatulence occurred following consumption of all ONS formulas: original formula (*n* = 3; 17.65%), TRM (*n* = 1; 5.88%), and TRM30 (*n* = 3; 17.65%). The intensity of symptoms was reported as “mild” for all formulas; no participants experienced moderate or severe GI disturbance. The mean stool form following ONS consumption was reported as normal (scale 3–4), as indicated by the Bristol Stool Scale at 3.94 ± 0.23, 3.82 ± 0.15, and 4.47 ± 0.17 for TRM30, TRM15, and original formula, respectively.

In Phase I, RMD30 was selected to be used in the long-term study since it decreased the postprandial insulin response in healthy subjects. Postprandial hyperinsulinemia has been associated with several non-communicable diseases (NCDs), including obesity, diabetes mellitus, and cardiovascular diseases [28]. Therefore, food with lower insulin index is expected to lower the risk of NCDs.

### 3.2. Phase 2: Long-Term (12 Weeks) Effect of TM30 on Blood Lipid Profile, Gastrointestinal (GI) Tolerability, and Dietary Intake

#### 3.2.1. Baseline Characteristics

Twenty-two participants completed the study. Most of the participants were female (59.1%) with a mean age of 30.86 ± 1.76 years. In this phase, participants were stratified into two subgroups based on their baseline FPG profile: prediabetes (FPG 100–125 mg/dL or HbA1C between 5.7–6.4%) and normoglycemic (FPG < 100 mg/dL or HbA1C < 5.7%) [29]. Based on those classifications, 9 participants were classified as prediabetes and 13 participants were normoglycemic.

Four participants in the prediabetes group had FPG less than 100 mg/dL, yet their HbA1C was between 5.7–6.4%. The mean baselines of FPG and HbA1C for the prediabetes group were 100.22 ± 2.44 mg/dL and 5.79 ± 0.06%, respectively. Meanwhile, baselines of FPG and HbA1C for the normoglycemic group were 86.31 ± 1.71 mg/dL and 5.31 ± 0.05%, respectively. The baseline fasting insulin in the prediabetes group and normoglycemic group was 12.61 ± 2.8 µIU/mL and 7.28 ± 0.99 µIU/mL, respectively (Table 3).

#### 3.2.2. Effect of ONS Supplementation on Metabolic Markers

Following 12-week intervention, FPG and insulin concentrations were not significantly altered. However, HbA1C results were significantly reduced by 5% and 5.8% in the prediabetic and normoglycemic groups (*p* < 0.001), respectively. Total cholesterol decreased insignificantly by 2.35% (from 198.33 ± 7.62 mg/dL to 193.67 ± 10.46 mg/dL) in the prediabetes group and 1.27% (from 193.31 ± 9.03 mg/dL to 190.85 ± 5.87 mg/dL) in the normoglycemic group. The reduction of total cholesterol was contributed by a reduction of LDL-cholesterol by 5.29% (from 132.33 ± 6.82 mg/dL to 125.33 ± 8.51 mg/dL) in the prediabetes group and 4.94% (from 121.46 ± 7.91 mg/dL to 115.46 ± 5.32 mg/dL) in the normoglycemic group. In contrast, HDL-cholesterol slightly increased by 3.21% (from 45.22 ± 3.51 mg/dL to 46.67 ± 2.74 mg/dL) in the prediabetes group and 3.78% (from 52.85 ± 4 mg/dL to 54.85 ± 3.62 mg/dL) in the normoglycemic group, respectively. Similarly, triglyceride also increased insignificantly by 2.78% in prediabetes and 8.14% in the normoglycemic group (*p* > 0.05). Liver enzymes, including AST and ALT, were not significantly altered. In addition, kidney enzymes, including BUN and creatinine, were similar between week 0 and week 12.

#### 3.2.3. Effect of ONS Supplementation on Body Composition and Food Intake

Body weight insignificantly increased with a mean change of 1.32 kg in the prediabetes group; however, body weight in the normoglycemic group remained stable between week 0 and week 12 (Table 4). The weight gain in the prediabetes group was caused by the increase of fat mass and muscle mass with mean change of 0.77 kg and 0.53 kg, respectively. Visceral fat rating significantly increased with a mean change of 0.78 in the prediabetes group, but not in the normoglycemic group. There was no significant difference in habitual energy, carbohydrate, fat, and protein intake following ONS supplementation for both groups (Table 5). Physical activity throughout 12 weeks of study was not significantly different.

#### 3.2.4. Effect of ONS Supplementation on GI Tolerability

There were no significant differences of GI symptoms throughout the 12-week supplementation period. However, mild bloating was reported by seven participants in the first week. Most of the participants defecated once or twice per day with normal shape of stool as indicated in No. 3–4 on the Bristol Stool Chart. Supplementation of ONS containing TRM for 12 weeks did not significantly affect the number of defecations per day and stool shape.

## 4. Discussion

This study demonstrates the beneficial effects of a novel ONS containing TRM (resistant starch) on postprandial plasma glucose and insulin responses without affecting subjective appetite, sensory acceptability, and GI tolerability. In addition, long-term (12 weeks) use of novel ONS contained 30% TRM reduced total cholesterol, LDL-cholesterol, and HbA1C in both prediabetes and normoglycemic participants; however, FBG was reduced only in prediabetic participants. There was no detrimental effect on liver and kidney functions following a long-term (12 weeks) use of ONS containing TRM 30%.

In this study, a single dose of 5.4 g TRM (30% replacement) insignificantly reduced the postprandial blood glucose response in healthy individuals. The result is in line with a previous study, which reported that an up to 50% replacement of TM by TRM did not significantly reduce the postprandial incremental plasma glucose [9]. However, a total replacement of TM by TRM decreased postprandial blood glucose by exerting an inhibition of amylase activity (in vitro) [9]. TRM may also diminish plasma glucose concentrations because of its α-1,2 and α-1,3 glycosidic linkage, which cannot be digested by human carbohydrate digestive enzymes [3]. The current study also proved the beneficial effect of (5.4 g) TRM on insulin response over 180 min. Similarly, Kishimoto et al. reported that cornstarch RM (5 and 10 g) significantly decreased the postprandial insulin response without altering glucose response when compared to a placebo following high fat meal consumption [6]. This finding indicates that less insulin is needed to control the postprandial blood glucose. The difference of the starch structure might influence the insulin response for healthy subjects [30]. Resistant starch lowers insulin response when compared to digestible starch since it is less susceptible for hydrolysis by amylolytic enzyme [30]. Therefore, resistant starch is digested slowly in the small intestine and, thus, decreases the glucose-dependent insulinotropic polypeptide (GIP) level as well as the insulin response. The decrease of GIP was correlated with a slower glucose clearance rate (GCR) following resistant starch consumption; therefore, following resistant starch consumption, the glucose response might not be significantly reduced, although insulin decreases [31]. However, further studies are necessary to investigate the underlying mechanism.

In our long-term study, a significant HbA1C reduction was observed following 12-week supplementation of ONS containing TRM in prediabetes and normoglycemic adults. It is believed that short-chain fatty acids (SCFAs) produced by a fermentation of TRM by gut microbiota induces glucagon-like peptide 1 (GLP-1) release and improves insulin release, thus lowering plasma glucose concentration [32]. HbA1C levels are reduced consequently.

Resistant maltodextrin has been known to inhibit lipid absorption in human and animal studies [6,33]. However, our long-term evaluation of ONS containing TRM found insignificant reduction of total cholesterol and LDL-cholesterol, while HDL-cholesterol and triglyceride were slightly increased. Similarly, a previous study showed that 24-week supplementation of high protein high fiber ONS decreased LDL-cholesterol by 3.25% (from 123 ± 37 mg/dL to 119 ± 36 mg/dL), while HDL-cholesterol increased by 10.26% (from 39 ± 10 mg/dL to 43 ± 10 mg/dL) [34]. Moderate improvement of lipid profile is beneficial to modulate cardiovascular disease (CVDs) risk [35,36]. It was noted that 1 mmol/L (1 mmol/L is equal to 38.67 mg/dL LDL-cholesterol) reduction in LDL-cholesterol decreases the relative risk of CVD events by 23% [35]. Thus, in this study, the addition of TRM to ONS may have produced beneficial effects regarding the risk of CVD events.

Resistant maltodextrin has been reported to prolong satiety in human [7,8,37,38]. The SCFAs resulting from fermentation of RM stimulates the release of gut hormones such as GLP-1 and peptide YY (PYY) that promotes satiety [8]. In contrast, this effect was not observed in this study as no significant differences in hunger, satiety, desire to eat, and prospective food consumption were found in all ONS formulas. It was hypothesized that either the dose or duration in this study was inadequate to have an effect. It takes approximately six to eight hours for RM to reach the colon and be fermented by gut microbiota [39]. In this study, we only observed subjective appetite for 180 min; therefore, the effect of SCFA fermentation may not have been observed. Similarly, Emilien et al. found that 10 to 20 g of RMD had an insignificant effect on subjective appetite over 150 min and there was no effect on food intake for the rest of the day following RM consumption [40]. Meanwhile, Ye et al. reported an increase of PYY and GLP-1 in accordance with the higher satiety response following 10 g consumption of RMD with a meal [8]. This insignificant results on prolong satiety may confirm the benefits of using TRM in ONS. Since the intentional use of ONS is to supplement energy and nutrient intake in addition to a normal diet, the satiety effect of ONS should be minimal to support the food intake for the next meal [41]. In long-term feeding, the habitual dietary intake was not affected by ONS supplementation throughout 12 weeks, which confirms that TRM containing did not alter the subjective appetite and habitual food intake in a long-term period. The body weights of normoglycemic participants were stable during intervention, while prediabetic participants insignificantly gained weight, which was correlated with the insulin level (data not shown). Insulin has been known to promote obesity by stimulating lipogenesis and inhibiting lipolysis [42]. Hommos et al. also showed that prediabetes adults increased more weight compared to normoglycemic adults in free-living conditions [43].

The replacements of 15% and 30% TM by TRM in ONS formulas were accepted as acceptable overall and rated > 7 (like moderately) on the 9-point hedonic scale compared with the original formula. In the current study, the viscosity of TRM30 was slightly lower when compared to the original formula (34.07 ± 0.09 vs. 36.37 ± 0.25 cP, *p* = <0.001) (unpublished work). A previous study revealed that less viscous ONS increased the ONS acceptability and consequently increased consumption by 33% [44]. Less viscous ONS was consumed faster and, thus, lowered the mouth-coating sensation without affecting satiety [44].

Fermentation of TRM by gut microbiota leads to increase gas production, including H_2_, CH_4_, and CO_2_, and that increases risk of GI discomfort [45]. Incorporation of TRM into ONS did not significantly affect the GI tolerability in both the acute and long-term study. After 12 weeks of ONS supplementation, mild gastrointestinal symptoms, including abdominal pain, bloating, vomiting, and flatulence were experienced in 5.88–17.65% of the participants after all ONS formulas. When comparing the three formulas, there was no significant difference in gastrointestinal symptoms. It indicated that TRM did not significantly affect the tolerability of ONS. Similarly, Mayr et al. showed that 14-day supplementation of ONS containing 2.5 g/100 mL from inulin and oat fiber was well tolerated in healthy subjects. In addition, the stool form following all formulas was categorized as the ideal stool form [46].

### Strengths and Limitations of the Study

In Phase I, the randomized cross-over controlled trial verified the results. This may have minimized the bias since subjects served as their own control. Furthermore, this study utilized TRM as a novel dietary fiber and evaluated its acute and long-term effect on metabolic profile in adults. However, there are some limitations of this study; the number of participants was too small in both phases. A larger sample size would be necessary to see significant effects in the results. Phase II was conducted during the COVID-19 pandemic, so control of physical activity was limited. In order to prove the benefits of TRM ONS on metabolic indicators, a study on the DM patients might be needed. This study did not investigate the underlying mechanism of TRM on satiety and glucose, including GLP-1, PYY, and SCFA production. Further studies are needed to clarify the mechanisms.

## 5. Conclusions

TRM, a novel non-viscous soluble resistant starch type IV, produced by partial enzymatic hydrolysis of tapioca starch leaves α,1-2, 1-3, 1-4, and 1-6 glycosidic linkage. With that structure, TRM is ingestible but fermentable in the human gastrointestinal tract. As a consequence, it was absorbed slowly and decreased insulin concentration without affecting satiety and GI tolerability. Long-term (12 weeks) use of TRM resulted in reduction of HbA1C in prediabetes and normoglycemic participants as well. A decrease in fasting plasma glucose in prediabetic participants was shown. The use of TRM for 12 weeks proved to be safe since there were no significant effects shown in the liver and kidney function enzyme tests. Together with the health benefits mentioned and its physicochemical characteristics being non-viscous, neutral in flavor and white in color, TRM has potential as a functional fiber in various food industries.

## Figures and Tables

**Table 1:** Macronutrient composition of the three oral nutritional supplements.

Ingredients	Original	TRM15	TRM30
Carbohydrate (g)	32.73	32.67	32.63
TRM (% of carbohydrate)	0.00	11.74	23.48
TM (% of carbohydrate)	78.26	66.52	54.78
Sucrose (% of carbohydrate)	21.74	21.74	21.74
Protein (g)	9.85	9.85	9.85
Whey protein isolate (% of protein)	49.11	49.11	49.11
Soy protein isolate (% of protein)	50.89	50.89	50.89
Fat (g)	9.05	9.05	9.05
Blended omega-3 oil powder (% of fat)	49.78	49.78	49.78
Rice bran creamer (% of fat)	49.78	49.78	49.78
Soy lecithin (% of fat)	0.45	0.45	0.45

TRM, tapioca-resistant maltodextrin. TM, tapioca maltodextrin.

**Table 2:** Baseline characteristics of participants in Phase I.

Baseline Characteristics	All Subjects (*n* = 17)
Gender (male/female)	7/10
Age (year)	26.24 ± 0.62
Body weight (kg)	56.05 ± 2.02
BMI (kg/m^2^)	21.29 ± 0.50
Fasting plasma glucose (mg/dL) Fasting insulin (µIU/mL)	91.62 ± 1.46 4.87 ± 0.68

BMI, body mass index.

**Table 3:** Blood chemistry at baseline and week 12 in Phase II.

Parameters	Pre-DM (*n* = 9)			Normal (*n* = 13)			All (*n* = 22)		
	Week 0	Week 12	*p*-Value	Week 0	Week 12	*p*-Value	Week 0	Week 12	*p*-Value
FBG (mg/dL)	100.22 ± 2.44	95.78 ± 4.02	0.216	86.31 ± 1.71	89.08 ± 1.76	0.175	92 ± 2.04	91.82 ± 2.02	0.924
HbA1C (%)	5.79 ± 0.06	5.5 ± 0.09	0.006 *	5.31 ± 0.05	5.00 ± 0.06	<0.001 *	5.5 ± 0.07	5.2 ± 0.07	<0.001 *
Insulin (µIU/mL)	12.61 ± 2.8	12.77 ± 2.47	0.917	7.28 ± 0.99	7.39 ± 1.09	0.942	9.46 ± 1.37	9.59 ± 1.3	0.931
Total cholesterol (mg/dL)	198.33 ± 7.62	193.67 ± 10.46	0.508	193.31 ± 9.03	190.85 ± 5.87	0.753	195.36 ± 6.07	192 ± 5.37	0.543
HDL	45.22 ± 3.51	46.67 ± 2.74	0.466	52.85 ± 4	54.85 ± 3.62	0.242	49.73 ± 2.83	51.5 ± 2.52	0.156
LDL	132.33 ± 6.82	125.33 ± 8.51	0.252	121.46 ± 7.91	115.46 ± 5.32	1.000	125.91 ± 5.45	119.5 ± 4.69	0.247
Triglyceride	104.11 ± 15.35	107 ± 18.81	0.857	94.46 ± 15.38	102.15 ± 12.22	0.196	98.41 ± 10.85	104.14 ± 10.28	0.385
AST	21 ± 2.39	22.22 ± 2.38	0.522	18.23 ± 1.6	21.46 ± 3.09	0.141	19.36 ± 1.36	21.77 ± 2.03	0.139
ALT	29.22 ± 5.01	35.11 ± 8.01	0.319	19.08 ± 2.98	18.69 ± 3.36	0.783	23.23 ± 2.84	25.41 ± 4.11	0.723
BUN	10.67 ± 0.5	10.78 ± 0.88	0.834	11.23 ± 1.2	10.04 ± 0.75	0.321	11 ± 0.73	10.34 ± 0.56	0.674
Creatinine	0.8 ± 0.05	0.79 ± 0.04	0.434	0.78 ± 0.05	0.79 ± 0.04	0.655	0.79 ± 0.03	0.79 ± 0.03	0.971
eGFR	110.78 ± 4.8	111.11 ± 3.92	0.831	111.15 ± 3.61	111.85 ± 3	0.827	111 ± 2.83	111.55 ± 2.33	0.776

* significant value less than 0.05. Data are presented as mean ± SEM

**Table 4:** Body composition at baseline and week 12 in Phase II.

Parameter	Pre-DM (*n* = 9)			Normal (*n* = 13)			All (*n* = 22)		
	Week 0	Week 12	*p*-Value	Week 0	Week 12	*p*-Value	Week 0	Week 12	*p*-Value
Body weight (kg)	69.84 ± 4.7	71.16 ± 4.65	0.128	62.26 ± 4.12	62.21 ± 3.96	0.907	65.36 ± 3.13	65.87 ± 3.1	0.252
BMI (kg/m^2^)	25.59 ± 1.1	26.1 ± 1.06	0.140	23.5 ± 1.15	23.52 ± 1.12	0.959	24.35 ± 0.83	24.58 ± 0.82	0.193
Fat (%)	28.51 ± 2.64	29.19 ± 2.55	0.381	27.17 ± 2.87	27.05 ± 2.65	0.869	27.72 ± 1.97	27.92 ± 1.86	0.699
Fat mass (kg)	20.11 ± 2.31	20.88 ± 2.2	0.303	17.26 ± 2.67	17.01 ± 2.44	0.944	18.43 ± 1.82	18.59 ± 1.72	0.417
FFM (kg)	49.73 ± 3.59	50.27 ± 3.59	0.201	45 ± 3.29	45.21 ± 3.27	0.593	46.94 ± 2.43	47.28 ± 2.43	0.211
Muscle mass (kg)	47.01 ± 3.44	47.54 ± 3.45	0.201	42.53 ± 3.13	42.74 ± 3.12	0.593	44.36 ± 2.32	44.7 ± 2.32	0.211
BMR (kcal)	1457 ± 95.24	1472 ± 94.81	0.184	1341.92 ± 86.48	1348.69 ± 86.49	0.463	1389 ± 63.96	1399.14 ± 64.04	0.139
Visceral fat rating	8.22 ± 1.3	9 ± 1.27	0.023 *	5.31 ± 1.02	5.15 ± 0.9	0.584	6.5 ± 0.84	6.73 ± 0.83	0.308

* Significant value less than 0.05. Data are presented as mean ± SEM.

**Table 5:** Habitual food intake of participants for 12 weeks.

Participants	Nutrient	Week 0	Week 3	Week 6	Week 9	Week 12	*p*-Value
All participants	Energy (kcal)	1103.99 ± 75.6	1086.98 ± 61.51	1164.97 ± 92.03	1189.08 ± 74.22	1182.67 ± 84.83	0.416
(*n* = 22)	Carbohydrate (g)	141.77 ± 9.28	134.54 ± 9.06	145.41 ± 11.74	149.76 ± 11.42	148.71 ± 11.41	0.539
	Fat (g)	39.28 ± 3.77	40.41 ± 3.26	43.05 ± 4.46	42.91 ± 3.05	42.75 ± 3.49	0.650
	Protein (g)	45.77 ± 3.77	48.3 ± 4.49	49.54 ± 4.13	50.91 ± 3.78	51.31 ± 4.28	0.502
Normal	Energy (kcal)	1075.93 ± 71.22	1050.54 ± 59.84	1109.91 ± 81.71	1158.42 ± 98.95	1173.24 ± 117.7	0.593
(*n* = 13)	Carbohydrate (g)	141.65 ± 11.87	130.82 ± 10.91	142.22 ± 10.91	145.26 ± 17.18	146.78 ± 15.59	0.629
	Fat (g)	36.73 ± 3.03	38.83 ± 4.33	38.95 ± 4.53	42.38 ± 3.07	41.91 ± 4.87	0.190
	Protein (g)	44.64 ± 4.09	45.12 ± 5.43	47.62 ± 3.98	48.99 ± 4.34	52.22 ± 6.46	0.247
Pre-DM	Energy (kcal)	1144.51 ± 159.17	1139.61 ± 126.22	1244.5 ± 196.31	1233.35 ± 117.12	1196.29 ± 126.96	0.728
(*n* = 9)	Carbohydrate (g)	141.94 ± 15.72	139.91 ± 16.2	150 ± 24.96	156.26 ± 13.74	151.51 ± 17.52	0.863
	Fat (g)	42.95 ± 8.28	42.69 ± 5.15	48.97 ± 8.71	43.67 ± 6.25	43.96 ± 5.15	0.525
	Protein (g)	47.41 ± 7.39	52.9 ± 7.79	52.3 ± 8.57	53.68 ± 7.01	50 ± 5.19	0.979

## Data Availability

All relevant data are within the paper.
